# Audit of ECG Monitoring in an Acute Mental Health Unit According to National Guidelines for Patients Receiving Anti-Psychotic medications

**DOI:** 10.1192/bjo.2026.11708

**Published:** 2026-06-30

**Authors:** Courtney Weir, Julie Black, Jonathan Hagan

**Affiliations:** Acute Mental Health Inpatient Centre, Belfast, United Kingdom

## Abstract

**Aims:**

Antipsychotic medicines can cause QT prolongation, are pro-arrhythmogenic (specifically, they can increase the risk of torsade de pointes) and are linked to sudden cardiac death. The Maudsley prescribing guidelines advise patients being treated with antipsychotic medications should have an ECG both on admission and before discharge if their medication regime has changed. The aim of this audit was to establish current adherence to national guidance in the Acute Mental Health Inpatient Centre (AMHIC) in the Belfast Health and Social Care Trust (BHSCT).

**Methods:**

The decision was made to collect data based on all the discharges from AMHIC. Data was retrospectively collected using the H&C numbers of the patients discharged in the entire month of December. The unit consists of 5 acute inpatient wards and in December there was a total of 42 discharges. Of these 42 patients, 8 were not on any antipsychotic medicines so they were discounted from the data set. A further 2 patients had no changes made to their antipsychotic during inpatient admission, so they were also not included. This left 34 patients on which to base the review. We collected information for 34 patients, including whether patients had an admission ECG, how long it took to get their admission ECG during admission and if this was being done in a ‘timely fashion’ (defined as within one week of admission for the purposes of this audit) and if patients got a discharge ECG.

**Results:**

100% of patients did not have a discharge ECG prior to them leaving hospital.

1. 20.59% (7/34) of the patients had no ECG completed during admission.

2. 50% (17/34) of patients did not have ECG completed within 7 days of admission to AMHIC.

**Conclusion:**

Results suggest that recommended ECG monitoring for patients on antipsychotic medicines is not being completed consistently at ward level. This has helped to assess areas for improvement and to put together plans for future interventions for ensuring patient safety while on antipsychotic medications. Interventions are required to improve timely ECGs both at admission and discharge within the inpatient setting. We have arranged a teaching session during the monthly patient safety meeting to communicate our findings to our medical and nursing colleagues. We have made reminder posters for each ward nurses’ station and clinical room. We plan to address this issue by using the PDSA model for effective change management.

